# The social determinants of mental health: new insights for mental health professionals

**DOI:** 10.1192/j.eurpsy.2025.37

**Published:** 2025-08-26

**Authors:** J. B. Kirkbride

**Affiliations:** Division of Psychiatry, UCL, London, United Kingdom

## Abstract

**Abstract:**

People exposed to more unfavourable social circumstances are more vulnerable to poor mental health over their life course, in ways that are often determined by structural factors which generate and perpetuate intergenerational cycles of disadvantage and poor health. Addressing these challenges has become an imperative matter of social justice. In this paper we provide a roadmap to address the social determinants that cause mental ill health. Relying as far as possible on high-quality evidence, we first map out the literature that supports a causal link between social determinants and later mental health outcomes. Given the breadth of this topic, we focus on the most pervasive social determinants across the life course, and those that are common across major mental disorders. We draw primarily on the available evidence from the Global North, acknowledging that other global contexts will face both similar and unique sets of social determinants that will require equitable attention. Much of our evidence focuses on mental health in groups who are marginalized, and thus often exposed to a multitude of intersecting social risk factors. These groups include refugees, asylum seekers and displaced persons, as well as ethnoracial minoritized groups; lesbian, gay, bisexual, transgender and queer (LGBTQ+) groups; and those living in poverty. We then introduce a preventive framework for conceptualizing the link between social determinants and mental health and disorder, which can guide much needed primary prevention strategies capable of reducing inequalities and improving population mental health. Following this, we provide a review of the evidence that has tested candidate preventive strategies to intervene on social determinants of mental health. These interventions fall broadly within the scope of universal, selected and indicated primary prevention strategies, but we also briefly review important secondary and tertiary strategies to promote recovery in those with existing mental disorders. Finally, we provide seven key recommendations, framed around social justice, which constitute a roadmap for action in research, policy and public health. Adoption of these recommendations would provide an opportunity to advance efforts to intervene on modifiable social determinants that affect population mental health.

**Disclosure of Interest:**

None Declared

